# Platelet miRNA Biosignature Discriminates between Dementia with Lewy Bodies and Alzheimer’s Disease

**DOI:** 10.3390/biomedicines9091272

**Published:** 2021-09-20

**Authors:** Ana Gámez-Valero, Jaume Campdelacreu, Dolores Vilas, Lourdes Ispierto, Jordi Gascón-Bayarri, Ramón Reñé, Ramiro Álvarez, Maria P. Armengol, Francesc E. Borràs, Katrin Beyer

**Affiliations:** 1 Department of Pathology, Germans Trias i Pujol Research Institute (IGTP), Universitat Autònoma de Barcelona (UAB), 08193 Barcelona, Spain; a.gamez@ub.edu; 2 REMAR-IVECAT Group, Germans Trias i Pujol Research Institute (IGTP), 08916 Badalona, Barcelona, Spain; 3 Servei de Neurologia, Hospital Universtiari Bellvitge, 08907 Hospitalet de Llobregat, Barcelona, Spain; jcampdelacreu@bellvitgehospital.cat (J.C.); jordigneuro@bellvitgehospital.cat (J.G.-B.); rrene@bellvitgehospital.cat (R.R.); 4 Servei de Neurologia, Hospital Universtiari Germans Trias i Pujol, 08916 Badalona, Barcelona, Spain; dvilas.germanstrias@gencat.cat (D.V.); mlispierto.germanstrias@gencat.cat (L.I.); ralvarez.germanstrias@gencat.cat (R.Á.); 5 Genomic and Microscopy Facilities, Germans Trias i Pujol Research Institute (IGTP), 08916 Badalona, Barcelona, Spain; mparmengol@igtp.cat; 6 Nephrology Service, Hospital Universitari Germans Trias i Pujol, 08916 Badalona, Barcelona, Spain

**Keywords:** Alzheimer disease, dementia with Lewy bodies, miRNA, peripheral biomarkers, platelets, synucleinopathies

## Abstract

Dementia with Lewy bodies (DLB) is one of the most common causes of degenerative dementia, after Alzheimer’s disease (AD), and presents pathological and clinical overlap with both AD and Parkinson’s disease (PD). Consequently, only one in three DLB cases is diagnosed correctly. Platelets, previously related to neurodegeneration, contain microRNAs (miRNAs) whose analysis may provide disease biomarkers. Here, we profiled the whole platelet miRNA transcriptome from DLB patients and healthy controls. Differentially expressed miRNAs were further validated in three consecutive studies from 2017 to 2019 enrolling 162 individuals, including DLB, AD, and PD patients, and healthy controls. Results comprised a seven-miRNA biosignature, showing the highest diagnostic potential for the differentiation between DLB and AD. Additionally, compared to controls, two miRNAs were down-regulated in DLB, four miRNAs were up-regulated in AD, and two miRNAs were down-regulated in PD. Predictive target analysis identified three disease-specific clusters of pathways as a result of platelet-miRNA deregulation. Our cross-sectional study assesses the identification of a novel, highly specific and sensitive platelet-associated miRNA-based biosignature, which distinguishes DLB from AD.

## 1. Introduction

Dementia with Lewy bodies (DLB) is the second most common cause of degenerative dementia after Alzheimer’s disease (AD) and, together with Parkinson’s disease (PD), it belongs to the group of Lewy body disorders (LBD) [1,2]. Besides Lewy body pathology, a high percentage of DLB brains contain concomitant AD pathology [3], also leading to the clinical overlap between DLB and AD. Although advances in the field have allowed improvements in their clinical characterization, it is still a challenge to diagnose DLB, AD and PD early and accurately [2]. In particular, up to 80% of DLB cases are still misdiagnosed, usually as AD, and patients receive treatments that can adversely affect their cognition [4]. Therefore, the identification of biomarkers that permit the differential diagnosis of these diseases is of paramount importance. Reduced Aβ42 levels have been found in CSF from AD patients [5], and tau and neurofilament are elevated in AD plasma and CSF compared with controls [6]. Although classical AD CSF-based biomarkers have been explored as potential differential diagnosis tools in DLB patients, results from various studies are controversial [7], and no peripheral biomarkers that differentiate between DLB and AD have been identified so far.

The study of blood and blood components has led to the identification of numerous circulating biomarkers. In particular, platelets are released into the circulation from the bone marrow after megakaryocytic differentiation. Despite being anucleate cells, platelets contain endoplasmic reticulum, ribosomes, and complete mitochondrial and apoptotic systems [8]. Additionally, a broad spectrum of functional mRNAs is found in platelets, which are translated after platelet activation. Since there are at least three different activation mechanisms, the resulting protein secretion profile depends on the specific activation pathway [9,10]. Platelets are, therefore, able to modify their proteome in response to different environmental changes and stimuli [11]. Regarding this specific gene-expression regulation, the presence of microRNAs (miRNAs) in platelets was described for the first time in 2008 [12], and Landry and colleagues confirmed the existence of an almost complete and functional miRNA pathway one year later [13]. Since then, several studies have been performed on the platelet miRNA content [14,15]. In addition to their role in haemostasis and thrombosis, the functions of platelets include induction of apoptosis, initiation of immune response and tissue remodelling [11]. They have also been described to show an enzymatic pathway similar to dopaminergic neurons [8], and they can store and release neurotransmitters, such as serotonin, glutamate and dopamine [16]. In AD, oxidative stress induces mitochondrial dysfunction and cell death in both platelets and neurons [17,18]. Additionally, platelets contain up to 95% of the circulating form of the amyloid precursor protein [18], they express several neuronal receptors and inflammatory-signalling molecules [8], and also contain α-synuclein [19]. Recently, it was also shown that platelets play an active role during adult neurogenesis in the hippocampus [20]. Finally, changes in activation, aggregation and morphology of platelets have been reported in PD, DLB, amyotrophic lateral sclerosis and multiple sclerosis [21].

In this context, the aim of this study was to find out if platelet miRNAs may represent suitable biomarkers for DLB, distinguishing it from controls, and also from AD. First, we wanted to examine the complete platelet miRNA content in DLB compared to healthy individuals. Afterwards, to know if the detected differences were also detectable in independent cohorts and if these profiles were disease-specific, we performed three independent validation studies, also including AD and PD patients. As a result, several sets of miRNAs differentiated DLB from the other groups of study subjects.

## 2. Materials and Methods

**Figure 1:**
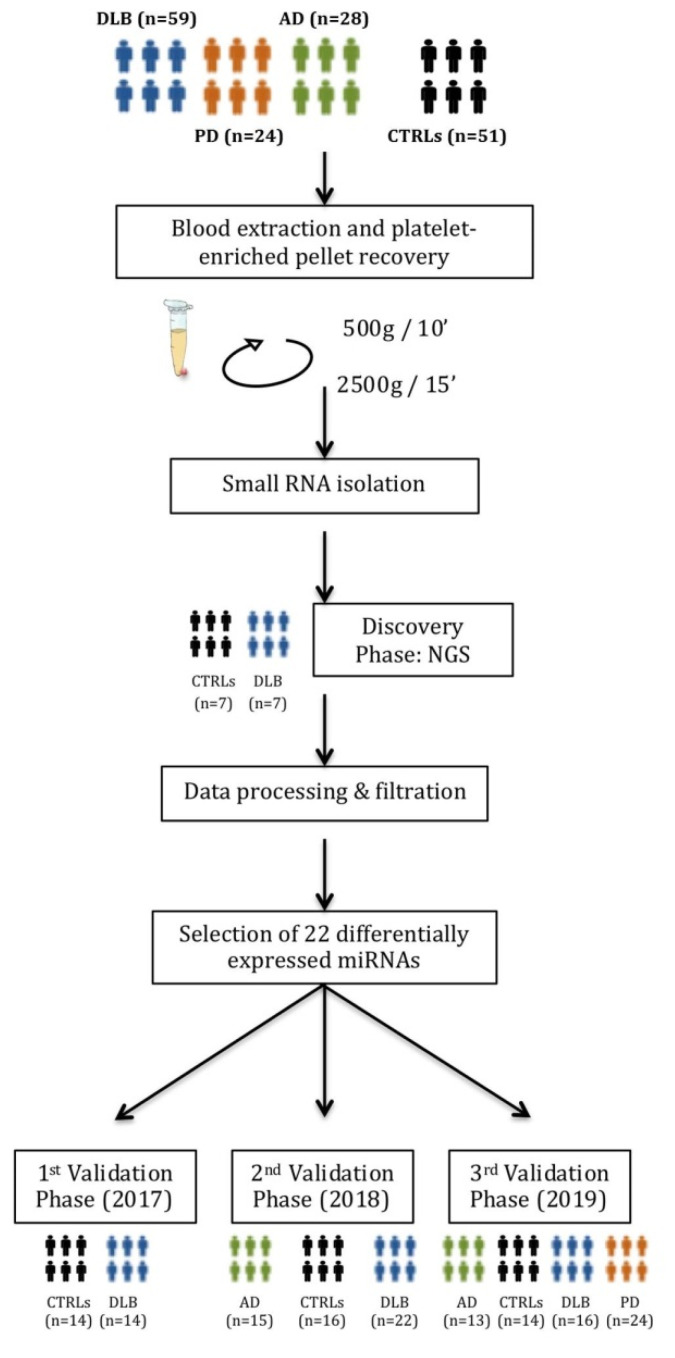
The complete workflow of the current study. A first discovery phase by Next-Generation Sequencing (NGS) included a cohort of 7 DLB patients and 7 healthy controls. Selected miRNAs were validated in three independent qPCR validation studies.

The whole workflow of this study is shown in Figure 1.

### 2.1. Participants

The current study was conducted between 2015 and 2019. A total of 162 individuals were included from two different hospitals: Hospital Universitari Germans Trias i Pujol (Badalona, Barcelona, Spain) and Hospital Universitari de Bellvitge (L’Hospitalet de Llobregat, Barcelona, Spain). Four cohorts were recruited:

DLB patients. Fifty-nine patients who fulfilled criteria for probable DLB [4,22] were prospectively recruited from those visited in the Neurodegenerative Disease Unit of both centres as routine clinical practice.

AD patients. Twenty-eight patients who fulfilled criteria for probable AD (National Institute on Aging–Alzheimer’s Association criteria) [23] were also consecutively recruited at the Neurodegenerative Disease Unit at their routine visits, irrespective of any specific complaint or clinical feature. AD patients were matched by age with the DLB patients.

PD patients. For comparison purposes, a group of 24 PD patients diagnosed according to the UK PD Society Brain Bank criteria [24] were included. None of these patients presented cognitive impairment, which was defined as subjective cognitive complaints, based on the patient’s and informant interview, and on the Minimental State Examination (MMSE) score, considering cognitive impairment if the MMSE punctuation was <24 points.

In DLB, AD and PD patients, age at onset was defined as the age when memory loss or parkinsonism was first noticed by the patient or his/her relatives.

Control subjects. Fifty-one control individuals were selected among non-blood relatives of the patients, age-matched with the DLB group.

The study was carried out in three independent phases; the first in 2017 included 21 DLB patients and 21 controls, the second in 2018 comprised 22 DLB, and 15 AD patients, and 16 controls, and the third in 2019 contained 16 DLB, 13 AD and 24 PD patients, and 14 controls.

The study was carried out with the approval of the local Ethical Committees for Clinical Investigation of the institutions involved in the study, and a written informed consent was signed by all participants or their legal guardians according to the Declaration of Helsinki [25].

### 2.2. Blood Collection, Purification and Characterization of Platelets

Peripheral blood was collected following standard procedures to minimize coagulation and platelet activation [26]. After venous puncture, blood was collected in sodium citrate tubes (BD Vacutainer^®^, Franklin Lakes, NJ, USA), and processed within 2 h following collection. After centrifugation at 500× *g* for 10 min at room temperature to pellet red blood cells and leukocytes, the supernatant was centrifuged at 2500× *g* for 15 min at room temperature to obtain a platelet-rich pellet [27]. The pellet was re-suspended in 250 µL of PBS and characterized by flow cytometry for sample purity according to typical platelet size and complexity (FSC/SSC) using 100 um-Red Nile Beads (ThermoFisher, Waltham, MA, USA) as reference and phenotypically confirmed as CD45−/CD61+ (ImmunoTools, Friesoythe, Germany; ref21270456 and ref21330613, respectively). The analysis was performed on a FACSCanto II flow cytometer (BD).

The samples were stored at −80 °C until further processing.

### 2.3. Purification of Platelet-Derived Small RNA

Platelet-rich pellets were thawed on ice. miRNA isolation was performed using the mirVana Paris Kit (Invitrogen, Carlsbad, CA, USA). Briefly, 600 µL of lysis buffer and 1/10 of miRNA Homogenate Additive Mix were added to each pellet and incubated for 10 min on ice after vortexing. One volume of phenol-chloroform was added, mixed and centrifuged at 10,000× *g* for 5 min. One-third and two-thirds volume of ethanol was added in 2 consecutive steps to the miRNA containing aqueous phase and passed through a filter column. After the recommended washing steps, miRNAs were obtained with 100 µL of elution buffer and stored at −80 °C until later analysis.

### 2.4. MiRNA Isolation from Whole Blood

RNA isolation was carried out after collection of 3 mL of whole blood in PAXgene Blood RNA tubes (PreAnalytiX, Hombrechtikon, Switzerland) with the PAXgene Blood miRNA Kit 50, v2 (PreAnalytiX) following manufacturer’s instructions. RNA concentration, purity and integrity were ascertained by the Agilent 2100 Bioanalyzer (Agilent Technologies, Santa Clara, CA, USA).

### 2.5. Discovery Phase: miRNA Sequencing and Sequencing Data Analysis

The total miRNA volume obtained from 7 DLB and 7 control samples was precipitated overnight at −20°C with 1 µL of glycogen (20 µg/µL), 10% 3 M AcNa (ph 4.8) and 2 volumes of ethanol. miRNAs were resuspended in 10 µL RNase free H_2_O and heated at 65 °C for 3 min. Quality control and size distribution of the purified small RNA was assessed by Bioanalyzer 2100 (Agilent Technologies, Santa Clara, CA, USA).

Six µL of each sample were used for library preparation with the NEBNext Multiplex Small RNA Sample Preparation Set for Illumina (New England Biolabs, Ipswich, MA, USA) following the manufacturer’s instructions. Individual libraries were subjected to quality analysis using a D1000 ScreenTape (TapeStation, Agilent Technologies, Santa Clara, CA, USA), quantified by fluorometry and pooled. Clustering and sequencing were performed on an Illumina Sequencer (MiSeq, Illumina, San Diego, CA, USA) at 1 × 50c single read mode, and 200,000 reads were obtained for each sample.

FastQ raw data obtained from the Illumina Platform were analysed as follows. After removing adapter sequences from the reads obtained by Trimmomatic [28], reads were mapped to the genome sequence using the Bowtie2 algorithm [29]. For each sample, miRNAs were identified, and the number of reads matching with a particular miRNA sequence was counted. The final count matrix was normalised through the weighted trimmed means of M-values (TMM) [30]. Putative biomarkers were selected following several criteria: (a) minimum of 5 reads per sample; (b) present in all patient samples and absent (less than 5 reads) in more than half of the control samples; (c) present in all control samples and absent in more than the half of the patient samples. In all cases, and when a miRNA was qualitatively present in both cohorts, differential expression analyses were carried out applying the Lilliefors’ composite goodness-of-fit test, Jarque-Bera hypothesis test and Shapiro–Wilk test to test for normal distributions. The Wilcoxon-rank sum test (*p*-value < 0.05) was used to determine whether miRNAs were differentially expressed between both cohorts. Two different concepts were considered for the selection of putative biomarkers and further validation: differentially expressed miRNAs (Wilcoxon-rank *p* < 0.05) and miRNAs with a good classifier potential [31]. A classifier is defined as the discriminant function that allows correctly classifying the types of database samples by using one or more independent variables. The methods tested were GLM binomial [32] and naive Bayes [33]. The validation process of the obtained differences was analysed by Leave-One-Out (LOO) cross-validation.

The raw and normalized data of this study have been deposited (accessed on 18 March 2020) at ncbi.nlm.nih.gov/geo/query/acc.cgi?acc=GSE147218 and https://www.ncbi.nlm.nih.gov/sra/, BioProject-ID: PRJNA613191.

### 2.6. Validation Phase: Reverse Transcription and Quantitative Real-Time PCR

MiRNA was reverse-transcribed using the MiRCURY LNA^TM^ Universal cDNA Synthesis Kit II (Exiqon, Vedbaek, Denmark) according to the manufacturer’s protocol. After adjusting RNA concentration to 5 ng/µL and mixing with reaction buffer and enzyme mix, a retro-transcription reaction was carried out at 42 °C for 60 min. Artificial RNA UniSp6 from the same kit was used as a retro-transcription control. Quantitative PCR (qPCR) was performed on a LightCycler 480 (Roche, Basel, Switzerland) using miRNA LNA technology and Pick&Mix PCR pre-designed panels (Exiqon, Vedbaek, Denmark) with miRNA UniSp3 as interplate calibrator control. cDNA was diluted 1:80, 4 µL were used with ExiLENT SYBR Green Master Mix (Exiqon, Vedbaek, Denmark) following manufacturer’s indications and samples were set up in duplicate.

The validation study was carried out in three independent phases, the first in 2017, the second in 2018 and the third in 2019, including the subjects as described in Section 2.1.

### 2.7. Statistical Analysis

Values for NGS data and reads are given as mean ± SD. Expression levels of the miRNAs selected for qPCR validation were determined using crossing point (Cp) values. Cp values were averaged between duplicates and normalized against UniSp6 spike-in Cp values for platelet-derived miRNA and against hsa-miR-191-5p in the case of whole blood. Relative expression changes were calculated by the −ΔΔCt method [34] and the results were further evaluated with the Wilcoxon–Mann–Whitney test (https://ccb-compute2.cs.uni-saarland.de/wtest/ access on 21 March 2020) and the two-tailed unpaired *t*-test to compare the expression between two groups. When comparing more than two groups (DLB, controls, AD and PD), multiple comparisons were performed using the Kruskal–Wallis non-parametric test and Dunn’s test was used for multiple corrections (GraphPad Software, Inc., La Jolla, CA, USA). In all cases, a confidence interval of 95% and a *p*-value below 0.05 was considered to be significant. To assess the diagnostic potential, the area under the ROC curve (AUC) was calculated for miRNAs with *p*-value < 0.01 by the Wilson/Brown method using SPSS Statistics 21 (IBM, Armonk, NY, USA) and GraphPadPrism v7 in order to determine the diagnostic sensitivity and specificity (95% C.I., AUC > 0.80 was considered as the minimum value for a useful biomarker).

### 2.8. miRNA Target Prediction and Analysis

Possible targets of deregulated miRNAs (those, showing *p*-value < 0.01) were predicted using miRTarbase [35] accepting as target genes those that were reported only by strong evidence studies; and by miRGate [36], considering only confirmed targets. For each miRNA set, including miRNAs with expression change of *p* < 0.01 and identified as disease-specific, targets from both databases were taken together, and overlapping data were removed before screening the complete list for their molecular relationship with String DataBase [37] and the Reactome online tool [38]. Gene description and most relevant information were screened also through the Uniprot database [39]. For each miRNA set, target genes were clusters by their functional characteristics, and related molecular pathway.

## 3. Results

### 3.1. Demographic and Clinical Data

Demographic and clinical data of patients are shown in Table 1. Mean age was similar between DLB patients, AD patients and controls, according to the inclusion criteria (75.1 #xB1; 6.8 years in the DLB group, 73.9 #xB1; 6.7 years in the AD group; *p* = 0.236); however, PD patients were significantly younger (66.9 #xB1; 14.9 years, *p* = 0.021). The male–female ratio was higher in PD and DLB than in AD and CTRLs, but no gender-specific expression changes were observed during data analyses. Disease duration was similar between patient groups (*p* = 0.068).

### 3.2. Platelet Characterization and miRNA Profile Discovery

**Figure 2:**
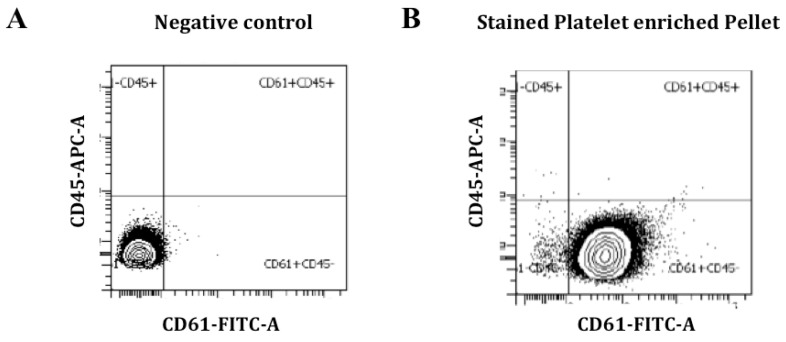
Platelet-rich pellet characterization by flow cytometry. CD61 staining was performed to identify platelets and CD45 was used as a leukocyte marker for staining of leukocyte contamination. Negative control with no CD61 staining (**A**); CD61-positive and CD45-negative staining is observed in the platelet-rich pellet (**B**).

Analysis of the platelet-rich pellet for possible leukocyte contamination showed no staining for the leukocyte marker CD45 in our samples. Instead, we obtained a high fluorescence signal for the platelet marker CD61, indicating high platelet purity and no leukocyte contamination (Figure 2).

**Figure 3:**
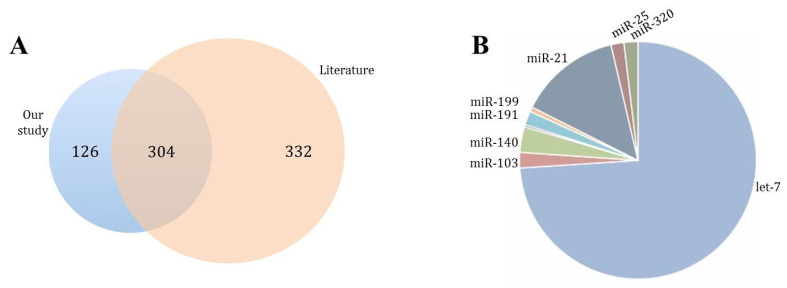
Bibliographic characterization of identified miRNAs. (**A**) Precursor-miRNA found in our study (blue circle) compared to literature (orange circle). (**B**) Most representative miRNA families found in our study.

RNA, used for the construction of NGS-libraries, showed an enriched profile of 20–40 nucleotide molecules characteristic for small RNA and miRNAs. The reads obtained by NGS were mapped onto 1279 different known mature miRNAs, and 534 miRNAs fulfilled the criterion of more than five reads per sample, corresponding to 430 different miRNA-precursors. The literature search revealed that 304 had been previously associated with platelets (Figure 3A), and 58.9% had been described in the first platelet-miRNA profiling studies [9,40]. Our study also confirmed let-7, miR-103 and miR-21 [15] as the most common platelet-miRNA families (Figure 3B).

The normalized counts from NGS data were analysed using the Wilcoxon-rank sum test and 11 miRNAs that were differentially expressed between DLB and controls, together with 11 miRNAs showing a good classifier potential were further validated by qPCR (hsa-miR-1343-3p, hsa-miR-191-3p, hsa-miR-6747-3p, hsa-miR-504-5p, hsa-miR-6741-3p, hsa-miR-128-3p, hsa-miR-1468-5p, hsa-miR-139-5p, hsa-let-7d-5p, hsa-let-7d-3p, hsa-miR-142-3p, hsa-miR-132-5p, hsa-miR-150-5p, hsa-miR-23a-5p, hsa-miR-26b-5p, hsa-miR-1301-3p, hsa-miR-625-3p, hsa-miR-146a-5p, hsa-miR-25-3p, hsa-miR-877-3p, hsa-miR-1908-5p, hsa-miR-744-5p) (Supplementary Table S1).

### 3.3. Validation of miRNA Expression

The 22 selected miRNAs were validated by qPCR in three independent studies.

#### 3.3.1. Study I (2017)

The first validation study included two cohorts of 21 DLB patients and 21 control individuals. Ten of the 22 miRNAs, hsa-miR-6747-3p, hsa-miR-128-3p, hsa-miR-139-5p, hsa-let-7d-5p, hsa-miR-142-3p, hsa-miR-132-5p, hsa-miR-150-5p, hsa-miR-26b-5p, hsa-miR-146a-5p, hsa-miR-25-3p, were diminished in DLB compared to controls (Table 2).

#### 3.3.2. Study II (2018)

Three independent cohorts comprising newly recruited DLB patients (n = 22), AD patients (n = 15) and control subjects (n = 16) were included in the second validation study. Although nine out of the ten miRNAs were again diminished in DLB when compared to controls, five out of these nine miRNAs failed to produce significant results due to an elevated intra-group variability. Yet, four miRNAs confirmed the results of Study I. miRNAs hsa-miR-128-3p, hsa-miR-139-5p, hsa-miR-150-5p, hsa-miR-25-3p, were significantly down-regulated in DLB compared to controls, with hsa-miR-150-5p showing the most important decrease (Table 3).

The comparison of DLB and AD miRNA expression data revealed that 9 out of the 10 miRNAs were significantly down-regulated in DLB compared with AD (Table 3). Only hsa-miR-150-5p was significantly diminished in AD when compared to controls.

#### 3.3.3. Study III (2019)

**Figure 4:**
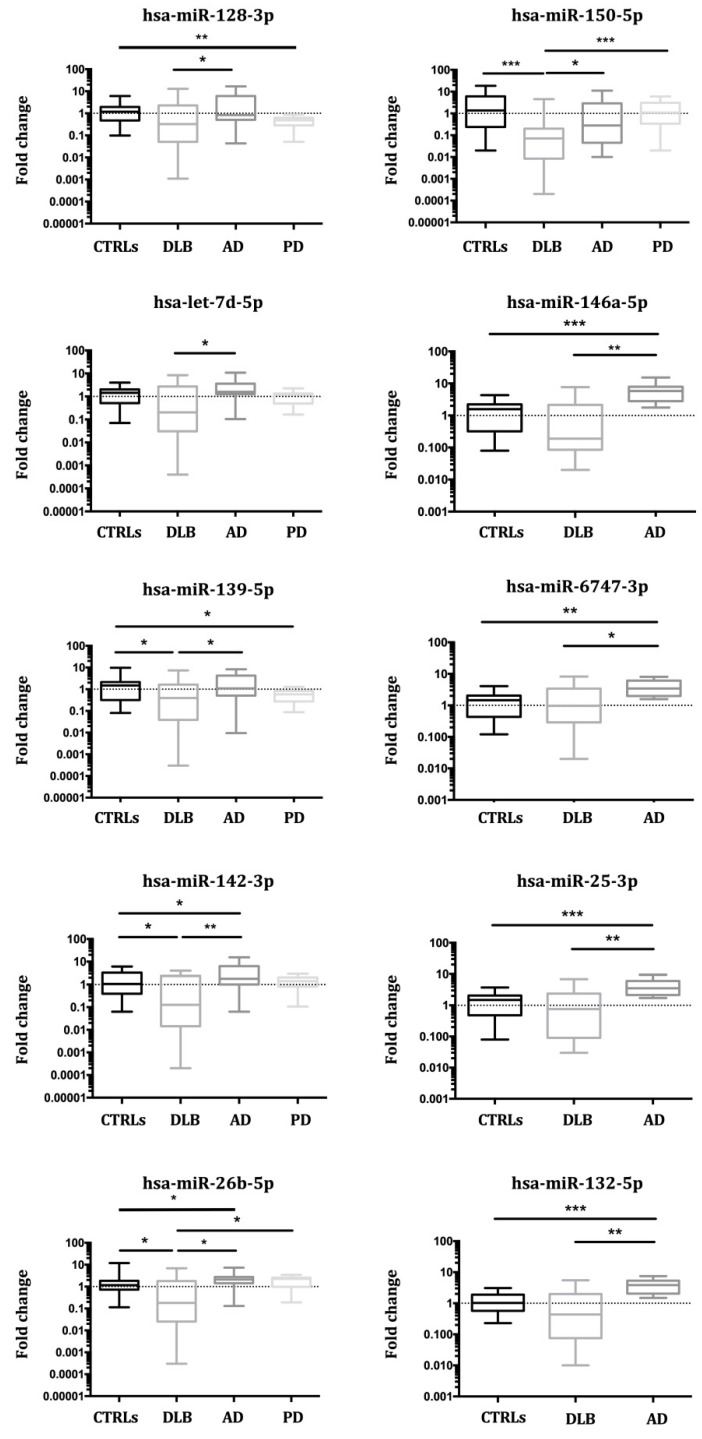
Combined results from three validation studies for miRNA expression in Controls, DLB, AD and PD. In all cases, mean and range for fold change are plotted; (* *p* < 0.05, ** *p* < 0.001, *** *p* < 0.0001).

To the initially recruited patients, four independent cohorts of newly diagnosed DLB (n = 16), AD (n = 13) and PD patients (n = 24), and 14 control subjects were added in the third validation study, analysing a total of 162 individuals (59 DLB patients, 28 AD patients, 24 PD patients, 51 controls). As a result, two miRNAs (hsa-miR-142-3p, hsa-miR-150-5p) were significantly diminished in DLB compared with controls. Seven miRNAs (hsa-let-7d-5p, hsa-miR-142-3p, hsa-miR-132-5p, hsa-miR-150-5p, hsa-miR-26b-5p, hsa-miR-146a-5p, hsa-miR-25-3p,) were significantly diminished in DLB compared to AD, and two (hsa-miR-150-5p and hsa-miR-26b-5p) were down-regulated in DLB compared to PD (Table 4). When grouping DLB and PD as LBD, only hsa-miR-139-5p was significantly down-regulated, but hsa-miR-128-3p and hsa-miR-139-5p were diminished in PD compared to controls. The expression of four miRNAs (hsa-miR-132-5p, hsa-miR-146a-5p, hsa-miR-25-3p, hsa-miR-6747-3p) was elevated in AD vs. CTRLs (Table 4, Figure 4).

**Figure 5:**
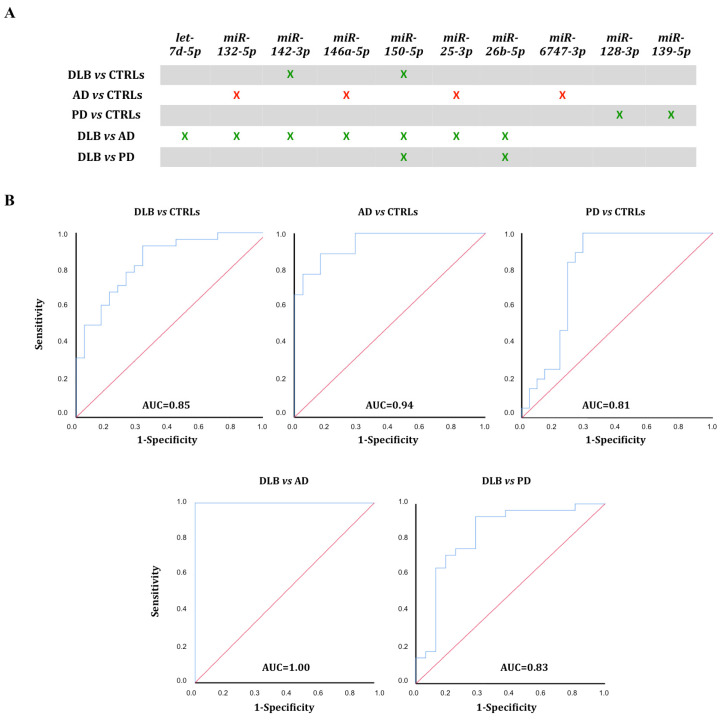
Diagnostic outcome for the five miRNA-sets. (**A**) Significantly different miRNAs (*p* < 0.01) were clustered into 5 different sets. Diminished expression—green, increased expression—red. (**B**) ROC curves were calculated for miRNAs with differential expression (*p* < 0.01) between two cohorts.

The five miRNA sets were further studied for their usefulness as biomarkers (Figure 5A).

### 3.4. ROC Curve Analysis

ROC curves were calculated for all five miRNA sets to assess their discrimination potential between groups. The combination of the seven differentially expressed miRNAs between DLB and AD (miRNAs hsa-let-7d-5p, hsa-miR-132-5p, hsa-miR-142-3p, hsa-miR-146a-5p, hsa-miR-150-5p, hsa-miR-25-3p and hsa-miR-26b-5p) presented the highest specificity (100%) and sensitivity (100%) to distinguish DLB patients from AD patients, with an AUC of 1 (Figure 5B).

The ROC curve for hsa-miR-142-3p and hsa-miR-150-5p, differentially expressed between DLB and CTRLs, yielded an AUC = 0.85 (95% C.I. 0.74–0.95; 82% sensitivity, 70% specificity). Comparison of AD and CTRLs, miRNAs hsa-miR-132-5p, hsa-miR-146a-5p, hsa-miR-25-3p, and hsa-miR-6747-3p resulted in AUC = 0.94 (95% C.I. 0.86–1.00; 89% sensitivity, 80% specificity); and AUC = 0.81 (95% C.I. 0.67–0.94; 84% sensitivity, 76% specificity) was obtained for hsa-miR-128-3p and hsa-miR-139-5p comparing PD and CTRLs. AUC = 0.83 (95% C.I. 0.73–0.98; 90% sensitivity, 73.7% specificity), was obtained for hsa-miR-150-5p and hsa-miR-26b-5p when comparing DLB and PD (Figure 5B).

### 3.5. miRNA Expression in Whole Blood

To assess whether the results were platelet-specific, we analysed the 10 differentially expressed miRNAs in whole blood of DLB, PD and AD patients, and controls (n = 16, each). Only the expression of hsa-let-7d-5p and hsa-miR-132-5p was diminished in blood of PD patients in comparison with controls. Specifically, these miRNAs did not show expression changes in platelets of PD patients. No additional differences were found (data not shown).

### 3.6. miRNA Target Prediction

To obtain a list of possible target genes, miRTarbase [35] and miRGate [36] were used to perform predictive analyses for the miRNAs sets with differential expression between DLB and CTRLs, DLB and AD, DLB and PD, AD and CTRLs, and PD and CTRLs.

**Figure 6:**
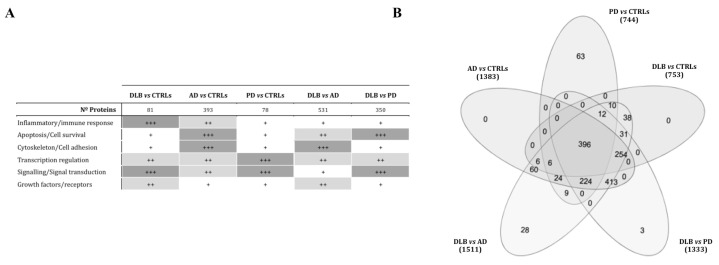
Target prediction analysis for the 5 miRNA sets was performed with miRTarbase and miRGate online tools. (**A**) Most relevant targeted pathways, according to Reactome and String analysis for each target-gene list. Mostly, genes related to transcriptional regulation and signal transduction were overrepresented (+++, ≥40% of the pathways related; ++, 20–40% of related pathways; +, up to 20% of the defined pathways). (**B**) Venn diagram comparing altered pathways in the 5 miRNA sets defined. In PD, 63 pathways were specifically altered, and 31 were associated with DLB in comparison to AD and PD.

When comparing DLB and controls, the target screening of miRNAs hsa-miR-150-5p and hsa-miR-142-3p identified 81 different genes. Genes involved in transcriptional regulation (*p* = 0.001), cellular response to stress (*p* = 8.36 × 10^−5^) and immune response (*p* = 0.021), were overrepresented (Figure 6A).

The seven miRNAs down-regulated in DLB compared to AD were predicted to target genes involved in integrin cell surface interactions (*p* = 1.5 × 10^−4^), cell death pathways (*p* = 0.002), and transcriptional regulation (*p* = 0.004) **(**Figure 6A). When analysing miRNAs diminished in DLB compared with PD, formation of senescence-associated heterochromatin foci (SAHF) was the most representative pathway (*p* = 2.4 × 10^−4^).

The analysis of the miRNA set that distinguished AD from controls (Figure 6A) rendered 393 possible target genes. Of these, 27.7% were involved in transcriptional regulation, 43.3% in signal transduction and 54.2% were phosphoprotein coding. Gene clusters related to stress (*p* = 8.3 × 10^−5^) and immune response (*p* = 1.58 × 10^−5^) were identified, including *TDRD7* (tudor-domain-containing protein 7) and *TIA1* (TIA1 cytotoxic granule associated RNA binding protein), both involved in stress granule formation.

MiRNAs hsa-miR-128-3p and hsa-miR-139-5p, down-regulated in PD compared to controls, were predicted to target 78 different genes. Signal transduction (*p* = 1.38 × 10^−10^) and PIP-AKT signalling (*p* = 4.9 × 10^−10^) were the most enriched pathways (Figure 6A). A protein-phosphorylation (*p* = 0.0376) and a MAPK-pathway (*p* = 0.015) cluster were identified including *FOS*, *MTOR* and *RICTOR* (Figure 6A).

Comparison of the five pathway lists revealed that 31 pathways were altered specifically in DLB (Figure 6B). RNA and small RNA metabolism (mitochondrial tRNA processing), and RNA silencing by small RNA were found. In AD, specific pathway enrichment comprised cell death-related pathways (*p* = 5.04 × 10^−8^). In PD, 63 pathways were specifically enriched (Figure 6B), including MAP kinase, protein phosphorylation pathways (*p* = 8.3 × 10^−8^), negative regulation of cell death (*p* = 0.009), and serotonin and dopamine receptor-related pathways (*p* = 0.05).

## 4. Discussion

In this study, we analysed the platelet miRNA profile in DLB patients, compared with AD and PD patients, and also with healthy controls, aiming to identify biomarkers for DLB. As a result of the first NGS discovery phase, we selected 22 differentially expressed miRNAs that were further validated in three independent qPCR-based studies, including independent cohorts of DLB, AD, PD, and controls. Since the clinical diagnosis of DLB is still challenging, primarily because of its overlap with AD but also with PD [4], there is an urgent need for biomarkers to differentiate between these neurodegenerative disorders. Here, we defined three different groups of miRNAs as being specifically deregulated in each of the three neurodegenerative diseases.

The first group was DLB-specific, consisted of seven miRNAs and comprising three subsets. Hsa-miR-142-3p and hsa-miR-150-5p showed diminished expression compared to controls; these two miRNAs, together with hsa-let-7d-5p, hsa-miR-132-5p, hsa-miR-146a-5p, hsa-miR-25-3p and hsa-miR-26b-5p were down-regulated compared to AD, and hsa-miR-150-5p and hsa-miR-26b-5p decreased in comparison with PD. Putative target genes were related to cell senescence, inflammation and signalling, and to RNA metabolism, especially to mitochondrial tRNA and gene silencing by small RNA. The disruption of RNA metabolism alterations in RNA splicing and processing, together with the deregulation of non-coding RNA has been described in several brain disorders [41]. In early PD brains, alterations in the small RNA profile are specifically related to tRNA fragments [42]. However, the relation between the impairment of these pathways and the development of DLB remains to be determined.

The second group, hsa-miR-132-5p, hsa-miR-146a-5p and hsa-miR-6747-3p, were up-regulated in AD. Among the predicted target genes, we found apoptosis and cell death, together with stress response-related genes. Specifically, *TIA1*, required for the formation of stress granules [43], and the gene codifying TDRD7, a component of cytoplasmic RNA granules [44], were identified. TIA1 is involved in RNA splicing and post-transcriptional gene regulation, has been found in stress granules [45], and has been related to tau oligomerization and neurofibrillary tangle deposition in AD [43]. Stress granules are formed in the cytoplasm during transient cellular stress, and their nature and biology could be altered in neurodegenerative diseases, with chronic and long-term stress [45].

The third group of miRNAs, hsa-miR-128-3p and hsa-miR-139-5p, were significantly decreased in PD. Although transcriptional regulation and signal transduction were enriched in all miRNA sets, these were importantly over-represented among the target genes for these two PD-specific deregulated miRNAs. Both *RICTOR* and *MTOR* are predicted targets for hsa-miR-128-3p and play an essential role in neuronal survival and synaptic plasticity [46]. In PD brains, MTOR expression and AKT functions are impaired [47]. MTOR over-expression impairs autophagy in genetic PD, enhancing α-synuclein deposition [48], and hsa-miR-128-3p down-regulation could be involved in the up-regulation of this pathway.

To our knowledge, the seven-miRNA biosignature composed of hsa-miR-142-3p, hsa-miR-150-5p, hsa-let-7d-5p, hsa-miR-132-5p, hsa-miR-146a-5p, hsa-miR-25-3p and hsa-miR-26b-5p represents the first molecular signature that permits to distinguish DLB from AD with high specificity and sensitivity. Although the precise involvement of these miRNAs in DLB pathology has yet to be clarified, the identification of these biomarker candidates is particularly important, because they may improve DLB diagnosis and correspondingly, patient management, treatment and outcome. The 4th consensus report of the DLB Consortium underlined the urgent need for developing guidelines and outcome measures for clinical trials in DLB [4], this study could be crucial in the identification of a diagnostic biomarker to define inclusion/exclusion criteria for either patients with DLB, PD or AD in clinical trials.

Interestingly, three disease-specific clusters of pathways and biological processes were identified as the result of platelet-miRNA deregulation. In DLB, pathways were related to gene expression and small RNA metabolism; in AD, to stress response; and in PD, to protein phosphorylation, metabolism and degradation. Since DLB and PD are synucleinopathies, the identification of rather similar pathways could have been expected. However, since none of the PD patients had developed dementia when the samples were obtained, the involvement of different pathways may reflect the mechanisms leading to early dementia development in synucleinopathies. The study of PD patients with dementia is needed to elucidate this question further.

No differences in miRNA expression were found in whole blood, indicating that platelet-specific miRNA deregulation could be related to disease pathogenesis. Since platelets present neuron-like metabolic pathways, previous studies have shown that in AD, APP acts as a platelet-membrane receptor contributing mostly to soluble β-amyloid after platelet activation [49]. Mitochondrial dysfunction, higher content in phosphorylated TDP43, and morphological and structural platelet changes in AD and PD have also been reported [50,51]. Whether miRNA deregulation in platelets promotes neurodegeneration or merely reflects its effects remains to be elucidated. However, a possible link between platelets and brain plasticity has been recently described [11], showing that platelets act directly on neural precursor cells in vitro, and that specific exercise-induced platelet activation leads to enhanced hippocampal neurogenesis [20].

Although this study was performed in a multi-centre setting, our results need to be replicated by independent studies in other laboratories, and in multi-national studies with larger cohorts. Further research should also address and confirm the alteration of the predicted biological pathways and their relationship with DLB, AD and PD. Additionally, these miRNAs should also be analysed in groups of individuals at risk of developing a synucleinopathy or dementia, as well as individuals with idiopathic REM sleep behaviour disorder (IRBD) and mild cognitive impairment (MCI).

## 5. Conclusions

In summary, two main findings must be highlighted. First, we showed that the miRNA content from platelets may represent a promising source of biomarkers. In particular, we defined a 7-miRNA biosignature that may represent a useful biomarker for the differentiation between DLB and AD patients. Second, we defined specific clusters of pathways and biological processes for DLB, AD and PD, underlining that the development of the different diseases is, at least in part, platelet driven, by affecting specific pathways.

## 6. Patents

The results of this study indicate that the seven-miRNA-signature might represent a valid biomarker for the differential diagnosis of DLB ruling out especially AD. Therefore, this miRNA signature has been protected by filing the PCT application “IN VITRO METHOD FOR THE DIAGNOSIS OF SYNUCLEINOPATHIES” with publication number WO2020016437 on 19 July 2019.

## Figures and Tables

**Table 1:** Demographic and clinical data of the participants of the study.

	DLB (n = 59)	AD (n = 28)	PD (n = 24)	CTRLs (n = 51)	*p* ^1^
Mean age, y ^2^ (age range, y)	75.1 (57–89)	73.9 (65–82)	66.9 (42–87)	72.0 (61–85)	0.0067
Gender (male/female ratio)	1.5:1	1:1.5	1.5:1	1:1.5	0.493
Disease duration ^3^, y (range, y)	4.2 (1.2–10.1)	3.4 (0.8–5.6)	5.2 (1.8–7.6)	-	0.068
MMSE ^4^, mean (range)	15.1 (3–28)	20.3 (6–28)	-	-	0.189
UPDRS-III ^5^, mean (range)	-	-	20.9 (5–39)	-	-
GDS ^6^, mean (range)	-	4.1 (3–6)	-	-	-
Parkinsonism, n (%)	47 (79.7%)	-	-	-	-
Positive DAT imaging, n (%)	55 (93.2%)	-	-	-	-

^1^ p, obtained by the Kruskal-Wallis test; ^2^ y, years; ^3^ from the beginning of cognitive symptoms for DLB and AD, and motor symptoms in the case of PD; ^4^ MMSE, Mini-Mental State Examination; ^5^ UPDRs-III, Unified Parkinson’s disease rating scale; ^6^ GDS, global deterioration scale.

**Table 2:** Expression changes of the 22 miRNAs identified as deregulated by NGS, in DLB versus controls. First validation study (2017).

miRNA	Expr Change ^1^	Dev Range ^2^	*p*-Value ^3^
1343-3p	0.31	0.11–0.88	0.24
191-3p	0.34	0.13–0.89	0.25
**6747-3p**	**0.33**	**0.14–0.74**	**0.033**
504-5p	0.6	0.55–0.66	0.25
6741-3p	0.32	0.16–0.66	0.17
**128-3p**	**0.17**	**0.03–0.71**	**0.039**
1468-5p	0.37	0.16–0.89	0.28
**139-5p**	**0.26**	**0.09–0.69**	**0.029**
**7d-5p**	**0.16**	**0.05–0.55**	**0.015**
7d-3p	0.34	0.12–0.95	0.21
**142-3p**	**0.11**	**0.03–0.47**	**0.015**
**132-5p**	**0.21**	**0.06–0.70**	**0.018**
**150-5p**	**0.03**	**0.02–0.04**	**<0.0001**
23a-5p	0.58	0.41–0.81	0.31
**26b-5p**	**0.16**	**0.04–0.62**	**0.017**
1301-3p	0.28	0.06–1.28	0.24
625-3p	0.32	0.07–0.41	0.14
**146a-5p**	**0.16**	**0.05–0.53**	**0.017**
**25-3p**	**0.20**	**0.06–0.66**	**0.034**
877-3p	0.34	0.14–0.84	0.22
1908-5p	0.30	0.09–094	0.21
744-5p	0.21	0.08–0.59	0.19

^1^ expr change, expression change obtained by the ΔΔCt method after comparing two groups; ^2^ dev range, deviation range of the expression change; ^3^ *p*-value obtained by the Wilcoxon–Mann–Whitney followed by Dunn’s test for multiple corrections. Significant results are highlighted in bold.

**Table 3:** MiRNA expression results: Study II.

miRNA	DLB vs. CTRLs	AD vs. CTRLs	DLB vs. AD
n	22 vs. 16	15 vs. 16	22 vs. 15
**let-7d-5p**			
expr change ^1^	0.21	1.40	**0.14**
dev range ^2^	0.024–1.73	1.12–1.75	**0.02–0.98**
*p*-value ^3^	0.20	0.19	**0.042**
**miR-128-3p**			
expr change	**0.16**	1.25	**0.13**
dev range	**0.013–2.07**	1.07–1.47	**0.012–1.41**
*p*-value	**0.043**	0.19	**0.009**
**miR-132-5p**			
expr change	0.23	1.50	**0.15**
dev range	0.041–1.21	1.49–1.51	**0.028–0.80**
*p*-value	0.14	0.072	**0.011**
**miR-139-5p**			
expr change	**0.18**	1.17	**0.15**
dev range	**0.019–1.68**	0.88–1.56	**0.022–1.08**
*p*-value	**0.03**	0.68	**0.014**
**miR-142-3p**			
expr change	0.16	1.73	**0.09**
dev range	0.02–1.26	1.48–2.02	**0.009–0.85**
*p*-value	0.05	0.07	**0.0026**
**miR-146a-5p**			
expr change	0.18	1.51	**0.12**
dev range	0.017–1.87	1.37–1.66	**0.012–1.12**
*p*-value	0.069	0.11	**0.0089**
**miR-150-5p**			
expr change	**0.011**	**0.34**	**0.031**
dev range	**0.003–0.034**	**0.29–0.40**	**0.008–0.118**
*p*-value	**<0.0001**	**0.029**	**<0.0001**
**miR-25-3p**			
expr change	**0.20**	1.15	**0.17**
dev range	**0.026–1.52**	0.94–1.41	**0.02–1.08**
*p*-value	**0.03**	0.85	**0.025**
**miR-26b-5p**			
expr change	0.19	1.59	**0.12**
dev range	0.02–1.83	1.49–1.70	**0.013–1.07**
*p*-value	0.092	0.075	**0.0071**
**miR-6747-3p**			
expr change	0.62	1.39	0.44
dev range	0.24–1.55	1.23–1.56	0.20–0.99
*p*-value	0.92	0.25	0.25

^1^ expr change, expression change obtained by the ΔΔCt method after comparing two groups; ^2^ dev range, deviation range of the expression change; ^3^ *p*-value obtained by the Wilcoxon–Mann–Whitney followed by Dunn’s test for multiple corrections. Significant results are highlighted in bold.

**Table 4:** Combined results of Studies I-III.

miRNA	DLB vs. CTRLs	AD vs. CTRLs	PD vs. CTRLs	DLB vs. AD	DLB vs. PD
n	59 vs. 51	28 vs. 51	24 vs. 51	59 vs. 28	59 vs. 24
**let-7d-5p**					
expr change ^1^	0.25	1.81	0.98	**0.14**	0.28
dev range ^2^	0.05–1.16	1.74–1.84	0.61–1.29	**0.03–0.62**	0.04–1.91
*p*-value ^3^	0.08	0.102	0.17	**0.006**	0.15
**miR-128-3p**					
expr change	0.26	1.38	**0.38**	0.19	0.87
dev range	0.05–1.45	0.79–2.4	**0.3–0.49**	0.06–0.61	0.69–1.13
*p*-value	0.106	0.57	**0.0007**	0.05	0.89
**miR-132-5p**					
expr change	0.43	**3.50**	0.58	**0.12**	0.89
dev range	0.15–0.81	**2.92–4.20**	0.27–0.97	**0.04–0.42**	0.76–1.01
*p*-value	0.201	**<0.0001**	0.18	**0.001**	0.81
**miR-139-5p**					
expr change	0.20	1.22	**0.48**	0.16	0.41
dev range	0.04–0.91	1.14–1.3	**0.30–0.78**	0.04–0.70	0.06–3.04
*p*-value	0.05	0.96	**0.008**	0.05	0.68
**miR-142-3p**					
expr change	**0.14**	2.09	1.10	**0.07**	0.12
dev range	**0.02–0.78**	1.99–2.21	0.80–1.52	**0.01–0.35**	0.02–0.97
*p*-value	**0.007**	0.05	0.65	**0.0005**	0.05
**miR-146a-5p**					
expr change	0.34	**5.00**	0.67	**0.07**	0.72
dev range	0.18–0.66	**3.03–8.27**	0.51–0.84	**0.02–0.22**	0.46–1.02
*p*-value	0.05	**0.00013**	0.41	**0.0004**	0.45
**miR-150-5p**					
expr change	**0.04**	0.36	0.87	**0.10**	**0.04**
dev range	**0.02–0.07**	0.28–0.46	0.49–1.53	**0.07–0.17**	**0.01–0.14**
*p*-value	**<0.0001**	0.1	0.69	**0.01**	**<0.0001**
**miR-25-3p**					
expr change	0.47	**3.76**	0.71	**0.13**	0.83
dev range	0.22–1.00	**2.38–5.93**	0.49–1.1	**0.04–0.42**	0.67–1.09
*p*-value	0.23	**0.0003**	0.76	**0.002**	0.49
**miR-26b-5p**					
expr change	0.18	1.65	1.54	**0.09**	**0.11**
dev range	0.03–1.07	1.12–1.80	1.24–1.92	**0.01–0.68**	**0.02–0.86**
*p*-value	0.05	0.05	0.05	**0.001**	**0.008**
**miR-6747-3p**					
expr change	0.76	**3.50**	1.23	0.22	0.56
dev range	0.35–1.63	**2.40–5.10**	1.02–1.46	0.07–0.68	0.52–0.64
*p*-value	0.71	**0.0006**	0.29	0.05	0.09

^1^ expr change, expression change obtained by the ΔΔCt method after comparing two groups; ^2^ dev range, deviation range of the expression change; ^3^ *p*-value obtained by the Wilcoxon–Mann–Whitney followed by Dunn’s test for multiple corrections. Significant results are highlighted in bold.

## Data Availability

The RNA-seq data were deposited in NCBI GEO database (https://www.ncbi.nlm.nih.gov/geo/, series GSE147218) and at SRA (https://www.ncbi.nlm.nih.gov/sra/, BioProject-ID: PRJNA613191).

## Supplementary Materials

The following are available online at https://www.mdpi.com/article/10.3390/biomedicines9091272/s1, Table S1: Differential expression analysis of RNA sequencing data obtained for DLB and control samples.
